# Antimicrobial Fibrous Bandage-like Scaffolds Using Clove Bud Oil

**DOI:** 10.3390/jfb13030136

**Published:** 2022-08-30

**Authors:** Carlota von Thadden, Esra Altun, Mehmet Aydogdu, Mohan Edirisinghe, Jubair Ahmed

**Affiliations:** Department of Mechanical Engineering, University College London, London WC1E 7JE, UK

**Keywords:** clove bud, anti-microbial, bandages, natural remedies, commercial production

## Abstract

Wounds are characterised by an anatomical disruption of the skin; this leaves the body exposed to opportunistic pathogens which contribute to infections. Current wound healing bandages do little to protect against this and when they do, they can often utilise harmful additions. Historically, plant-based constituents have been extensively used for wound treatment and are proven beneficial in such environments. In this work, the essential oil of clove bud (*Syzygium aromaticum*) was incorporated in a polycaprolactone (PCL) solution, and 44.4% (*v*/*v*) oil-containing fibres were produced through pressurised gyration. The antimicrobial activity of these bandage-like fibres was analysed using *in vitro* disk diffusion and the physical fibre properties were also assessed. The work showed that advantageous fibre morphologies were achieved with diameters of 10.90 ± 4.99 μm. The clove bud oil fibres demonstrated good antimicrobial properties. They exhibited inhibition zone diameters of 30, 18, 11, and 20 mm against microbial colonies of *C. albicans*, *E. coli*, *S. aureus*, and *S. pyogenes*, respectively. These microbial species are commonly problematic in environments where the skin barrier is compromised. The outcomes of this study are thus very promising and suggest that clove bud oil is highly suitable to be applied as a natural sustainable alternative to modern medicine.

## 1. Introduction

The usage of plants for medical purposes dates back thousands of years. The first written source stems from around 3000 BC, where 12 recipes for natural drug preparation were written on a Sumerian clay slab in Nagpur, India [1]. Over time, chemicals have replaced natural remedies. Nevertheless, discussions regarding the toxicity of these in the human body encourages us to revisit these natural sources. The side effects of commonly used modern drugs range from redness/irritation and allergies to drug resistance and organ damage; this can mostly be avoided by utilising plant-based treatments. In the modern era of sustainability and environmentally friendly living, this kind of approach is even more noteworthy and should be pursued with priority [2].

Many plant-based extracts that have various benefits that are able to promote cutaneous wound healing exist [3]. Asiaticoside for example, extracted from *Centella asiatica*, also known as the Asian pennywort, is able to promote epithelialisation in wound healing and facilitate collagen deposition [4]. Alcohol extractions of the plant *Wedelia trilobata* have been used to treat chronic wounds, luteolin the principal yellow dye compound obtained from this plant has been shown to have many wound healing benefits including immunomodulation [5]. Luteolin has been shown to inhibit the expression of proinflammatory cytokines [6]. Another plant-based source of medicinal properties comes from *Aloe vera*, this is a significant example of a source which has many contributing bioactive compounds that work together to provide a beneficial effect to wound healing. *Aloe vera* contains many compounds such as polysaccharides, saponins, glycosides, phytol, anthraquinones, and oleic acid, isolating only a single compound could lead to a possible reduction in beneficial bioactivity [7].

Clove buds stem from the aromatic flower of trees belonging to the Myrtaceae family, native to Southeast Asia [8]. Clove therefore belongs to the species *Syzygium aromaticum* and also to *Eugenia caryophyllata* [9]. Clove bud oil is commonly used for culinary purposes as a spice, but also is widely known for improving oral health and other medical applications [10]. Clove bud oil is produced through distillation of the dry flowers [11]. It is known to contain 72–90% of the compound eugenol, which gives it a characteristic smell [12]. Other studies on the antimicrobial activity of eugenol have been positive [13].

This study focuses on assessing the antimicrobial activity of clove bud oil fibres against one of the most common fungi, *Candida albicans* (*C. albicans*), as well as four commonly occurring pathogenic bacteria: *Escherichia coli* (*E. coli*), *Staphylococcus aureus* (*S. aureus*), *Pseudomonas aeruginosa* (*P. aeruginosa*), and *Streptococcus pyogenes* (*S. pyogenes*). The study analyses the effect against these microbes when contained in a bandage for wound-healing purposes.

*C. albicans* is a yeast originating from opportunistic pathogens which cause infections in humans with compromised immune systems [14]. *C. albicans* is largely found in the gut flora and is one of the most common causes of fungal infections, which results from its overgrowth [15]. It is mostly known to be the source of urinary tract infections (UTIs), genital yeast infection as well as oral thrush [16,17]. *E. coli* refers to a bacterium generally found in the gut flora of warm-blooded animals [18]. Infections are usually harmless, yet some strains can cause serious foodborne diseases (e.g., diarrhoea, vomiting, UTIs), obtained through food contamination [19].

The bacterium *S. aureus* forms part of the microbiota of the body, where it is commonly found in the upper respiratory tract or on the skin [20]. It is considered an opportunistic pathogen that penetrates broken skin (wounds). Negative effects include respiratory infections (e.g., sinusitis, pneumonia) to skin infections (e.g., wound infections, abscesses). It is commonly treated by means of antibiotics. *P. aeruginosa* is known as a multidrug-resistant bacterium, which can survive under harsh environmental conditions. As an opportunistic pathogen, it enters a compromised body and can be responsible for serious acute and chronic infections at different sites of the body, these include extensive skin tissue damage, pneumonia, and UTIs [21]. It is highly resistant to antibiotics which leads to individual treatment on a case-to-case basis. The bacterium *S. pyogenes* forms part of the skin microbiota. It can cause numerous infections such as acute pharyngitis (strep throat) and toxic shock syndrome (TSS). It can also lead to severe (life-threatening) skin and soft-tissue infections [22]. It generally begins with throat and skin infections, such as a strawberry-like rash. It is commonly treated by penicillin [23].

Plasters, bandages, and scaffolds are commonly applied items to cover wounds. The fibrous structure permits permeability, diffusion, and the exchange of gases, yet still protects the wound from external trauma. Most commercially available bandages, such as gauzes, are made out of woven cotton [24]. Some include a polymer film coating on both sides to prevent adhering to the wound [25]. During wound healing, the body restores the damaged dermal and epidermal tissues with renewed tissue. However, wounds enable easy entrance of microbials which complicate wound healing through incurring infections. Patients in hospitals are especially susceptible to infection following operation. Pathogenic organisms, such as *E. coli*, enter the body and create infections. *C. albicans* is considered one of the most common human pathogens causing infections in clinical settings [26]. Research in international hospitals have identified mortality rates linked to invasive candidiasis to be as high as 40–60% of annual preventable deaths [27]. Further studies on infections after surgical treatments concluded the predominant pathogens to be *S. aureus*, *P. aeruginosa*, and *E. coli* [28].

**Figure 1:**
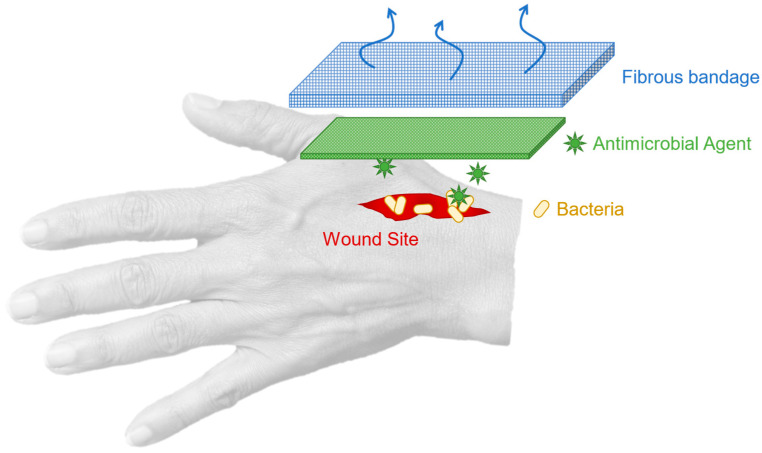
Concept and principles of applying antimicrobial dressings on wounds.

It is therefore useful to combine bandages with antimicrobial materials. A fibrous scaffold as a base for antimicrobial wound healing offers significant advantages, the structure permits the flow of air and filtration. Moreover, fibres with a low diameter increase the surface area-to-volume ratio, enabling efficient drug release, absorbency, and pliability [29]. A wound-healing bandage hence provides various benefits in assisting the healing process. The inserted antibacterial ingredients can actively fight bacterial growth if the wound is compromised. Figure 1 summarises the concept of applying an antimicrobial wound dressing to fight bacterial infections.

In 2013, the method of obtaining polymer fibres through pressurised gyration was invented at University College London (UCL) and later reviewed by Heseltine et al. [30]. It can be seen as a combination of solution blowing and centrifugal spinning; it is very scalable and simple to operate for mass production and manufacturing. The fibres generated through pressurised gyration can be used for medical applications such as tissue engineering and producing bandages [31,32,33,34]. Biodegradable polymers such as polycaprolactone offer biocompatibility and suitability in a range of biomedical applications from bone tissue engineering to wound healing, where pressurised gyration is ideal to produce fibrous meshes [35,36,37,38,39].

Current antimicrobial wound healing bandages commonly contain silver as the antibacterial agent [40]. However, silver dressings possess dose-dependent cytotoxicity to host cells such as epidermal keratinocytes and dermal fibroblasts [41]. The cytotoxic effect can be reduced through the exchange of silver with a more natural or plant-based antimicrobial drug. In this work, we present a novel approach to providing bandage-like materials which are sustainable because they come from natural sources, easy to produce as they incorporate a facile technology, and low environmental harm due to the use of natural oils. 

## 2. Materials and Methods

This work examined three essential oils: garlic, calendula, and clove buds. As both garlic and calendula oil showed no antimicrobial effect in their commercially obtainable form, only clove bud oil was studied in detail and incorporated into a fibrous bandage-like scaffold. This was achieved through the production of clove bud oil containing polycaprolactone (PCL) fibres by applying pressurised gyration. Clove bud essential oil (Naissance, Neath, UK) was outsourced and used for this research. 

**Figure 2:**
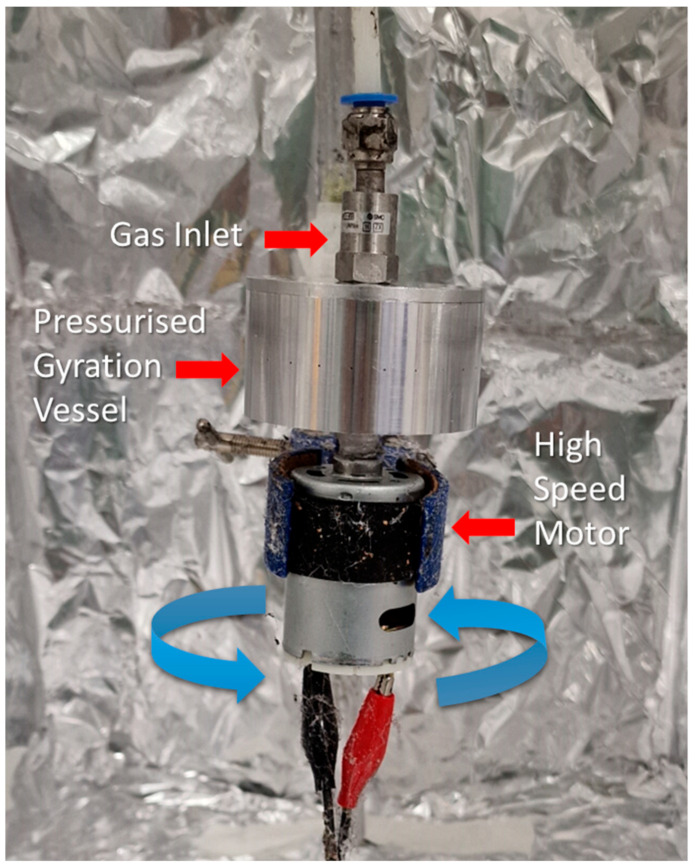
Schematic of the pressurised gyration setup, depicting the gas inlet, pressurised gyration vessel, and the high-speed rotation. The background is the surrounding chamber.

The laboratory setup of pressurised gyration consists of an aluminium cylindrical vessel (35 mm × 60 mm) containing 24 centred orifices, measuring 0.5 mm in diameter, through its wall. The vessel was connected on one side to a motor capable of rotation of up to 36,000 rpm. On the other side of the gyration vessel, a gas inlet was connected, which feeds in nitrogen gas; the applied pressure into the vessel can be controlled up to 0.3 MPa. A pre-prepared polymer solution was inserted into the vessel and spun at maximum speed, with an applied gas pressure of 0.1 MPa. The fibres were collected in the surrounding chamber. The schematic of the setup is depicted in Figure 2.

The principal driving force of the production is the centrifugal force, which is generated by the rotation of the high-speed motor; this leads to liquid extrusion through the vessel orifices once the centrifugal force exceeds the surface tension of the internal solution in the vessel. A polymer jet forms when the fluid is in a net positive equilibrium between viscous forces, surface tension, and dynamic fluid flow forces. The surface tension gradient appears along the liquid–air interface and focuses the polymer jet. This results in the occurrence of a Marangoni stress tangential to the liquid–gas interface elongating the advancing polymer droplet [42].

### 2.1. Fibre Preparation

Polymer solutions were prepared using the base (C0) of 15.5% *w*/*v* of PCL (Mn 80,000, Sigma Aldrich, Gillingham, UK) in Chloroform (CAS number: 67-66-3, Sigma Aldrich, Gillingham, UK). The clove bud oil used in this work was purchased from (Naissance, Neath, UK), which was a steam distilled 100% pure extraction of *Eugenia caryophyllus* originating from Indonesia. Three concentrations (C1, C2, and C3) of natural oils were examined. The concentrations of oil C1, C2, and C3 based on the weight of the PCL were 33.3%, 44.4%, and 50% respectively. To achieve homogenous solubility, the solutions were mixed for at least 12 h on a magnetic stirring plate. 

To produce the fibres, a volume of 2 mL for each case was inserted into the vessel of the pressurised gyration setup. The polymer solutions were then spun for 10 to 15 s at the maximum rotation speed, with an applied gas pressure of 0.1 MPa; this generated a rounded square of fibrous bandage-like scaffolds. All spinning was carried out under ambient conditions (~24 °C, ~45% relative humidity).

### 2.2. Anti-Microbial Testing

**Figure 3:**
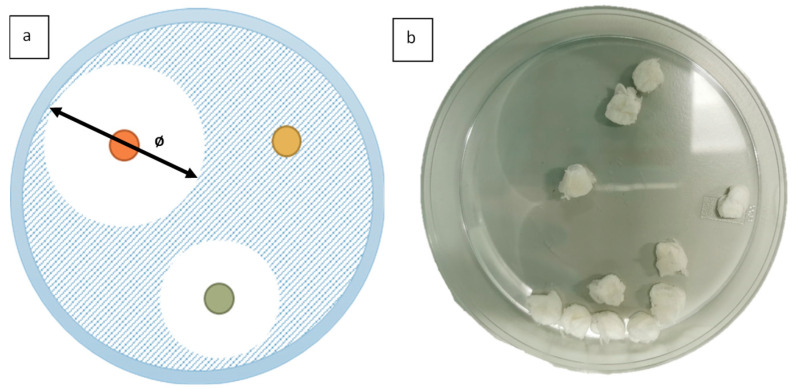
(**a**) Schematic of inhibition zone testing using inhibition zone diameter; (**b**) fibre samples prepared for antimicrobial testing (sample diameter 50 mm, weight 50 mg).

*In vitro* disk diffusion testing was chosen as the method to evaluate antimicrobial activity. The results of the tests are visible to the naked eye, and it is a common method that offers an easy and accessible comparison. Figure 3a shows a schematic of the visual result available when using inhibition zone testing.

Antimicrobial testing was carried out on C2 fibres containing clove bud oil, as concentrations exceeding 44.4% (*v*/*v*) clove bud oil/PCL would lead to inferior fibres being ultimately produced. This could be due to the interaction of clove bud oil with the PCL polymer chain in the dissolution process. Virgin PCL (C0) fibres were additionally analysed as a negative control. As a positive control, the oil was utilised by soaking in filter paper. All samples had a consistent diameter of 5 mm and a total mass of 50 mg. Figure 3b contains macro images of ten samples in a petri dish prepared for antimicrobial testing.

Petri dishes with Mueller–Hinton agar (Sigma Aldrich, Gillingham, UK) were prepared. One Petri dish would contain one fungus or bacteria colony and three samples. The fresh bacteria and fungi were diluted to ~10^7^ colony forming units. The bacteria and fungi were spread onto the agar plate using a sterile swab and the sample was situated onto the agar leaving space between each sample to observe the inhibition zone growth. The agar plates were incubated at an optimum temperature for 24 h. Through the use of the imaging software ImageJ (Version 1.53a, Madison, WI, USA), the diameter of the zone of inhibition, visible as a clear circle, was identified and measured in cm. A larger inhibition zone diameter correlates to higher antimicrobial activity. Percentages were used to represent the inhibition zone in comparison to the sample’s original diameter. For this, the inhibition zone was divided by 5 mm to obtain a percentage value. All tests were carried out in triplicate and the standard deviation is presented by error bars. Compared to the negative control (C0), all the clove bud oil-containing fibres showed a statistically significant increase in antimicrobial activity, except against *P. aeruginosa* when compared directly using paired *t*-tests with a *p* value of 0.05. 

## 3. Results and Discussion

**Figure 4:**
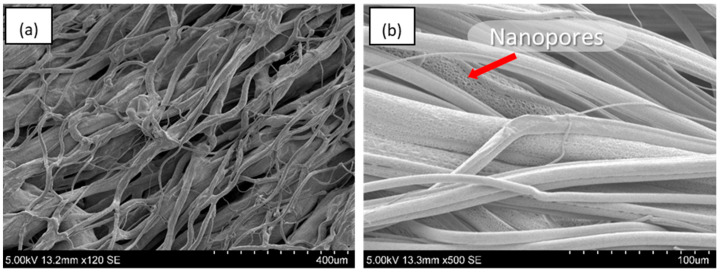
Scanning electron microscope images taken for gyration spun fibres (0.1 MPa) with the composition: (**a**) Virgin PCL fibres (C0); (**b**) clove bud oil fibres (C2). The presence of surface nanopores are indicated by an arrow.

The fibres containing C2 clove bud oil had an average fibre diameter of 10.90 ± 4.99 μm, comparable to the pure PCL fibres with a diameter of 10.87 ± 5.02 μm. This fibre diameter provides a sufficient surface area to volume ratio, permitting a higher contact area and improved diffusion between the antimicrobial bandage and wound site [43]. The surface morphology of these fibres appear to have a mixture of smooth surfaces and surfaces with the presence nanopores. These are the result of utilising a volatile solvent such as chloroform for the spinning process (Figure 4b) [44]. Surface pores can be beneficial in wound healing as it increases the available contact surface area and could provide filtration capabilities to the bandage.

Both the pure clove bud oil, as well as the clove bud oil fibres demonstrated significant antimicrobial activity on four of the five bacteria and fungi strains (*p* = 0.05). When compared to the size (ø 0.5 cm) and weight (50 mg) of the sample, the measured inhibition zone reveals very significant potential for the clove buds in biomedical applications. The largest inhibition zone was obtained against *C. albicans* (ø 3 ± 0.19 cm), followed by *S. pyogenes* (ø 2 ± 0.07 cm), *E. coli* (ø 1.8 ± 0.07 cm), and *S. aureus* (ø 1.1± 0.05 cm). There was no noticeable effect on *P. aeruginosa*.

### 3.1. Fourier-Transform Infrared Spectroscopy 

**Figure 5:**
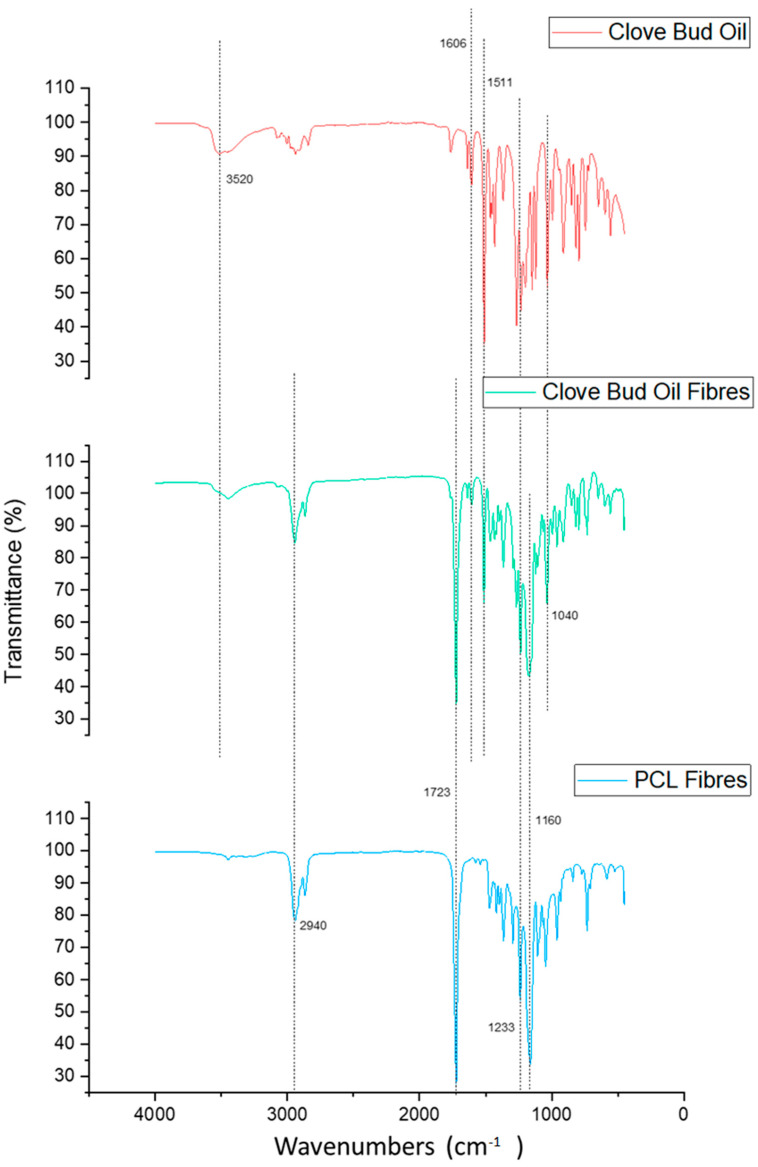
FTIR spectrum of clove bud oil fibres compared to the negative control virgin PCL fibres and the positive control pure clove bud oil.

After spinning the fibres, FTIR spectroscopy was utilised as a method to confirm the uptake of clove bud oil within the fibres. Virgin PCL (C0) fibres and all three fibres containing C1 concentration of the oils were tested, as well as the corresponding oils. The data obtained from the FTIR analysis were graphed together with the respective oil and the virgin PCL fibres. Figure 5 shows the comparison between the C1 clove bud oil fibres, the negative control virgin PCL fibres, and the positive control raw clove bud oil. The FTIR analysis was carried out at a wavelength range of 4000 to 400 cm^−1^.

The spectra of virgin PCL fibres (blue graph) accentuated characteristic peaks at wavenumbers of around 2940 (asymmetric CH2 stretching), 1723 (C=O stretching), 1233 (asymmetric C-O-C stretching), and 1160 cm^−1^ (symmetric C-O-C stretching). The wavenumbers and spectral bands match findings of electrospun PCL reported by Kuppan et al. and Liverani et al. [45,46]. 

The described spectral bands of PCL are also detected in the FTIR spectrum of clove bud oil fibres. The peaks confirm, as already known, the presence of PCL in the fibres. The difference can be linked to the presence of the oil, as the clove bud oil fibre spectrum lies between both spectra.

**Figure 6:**
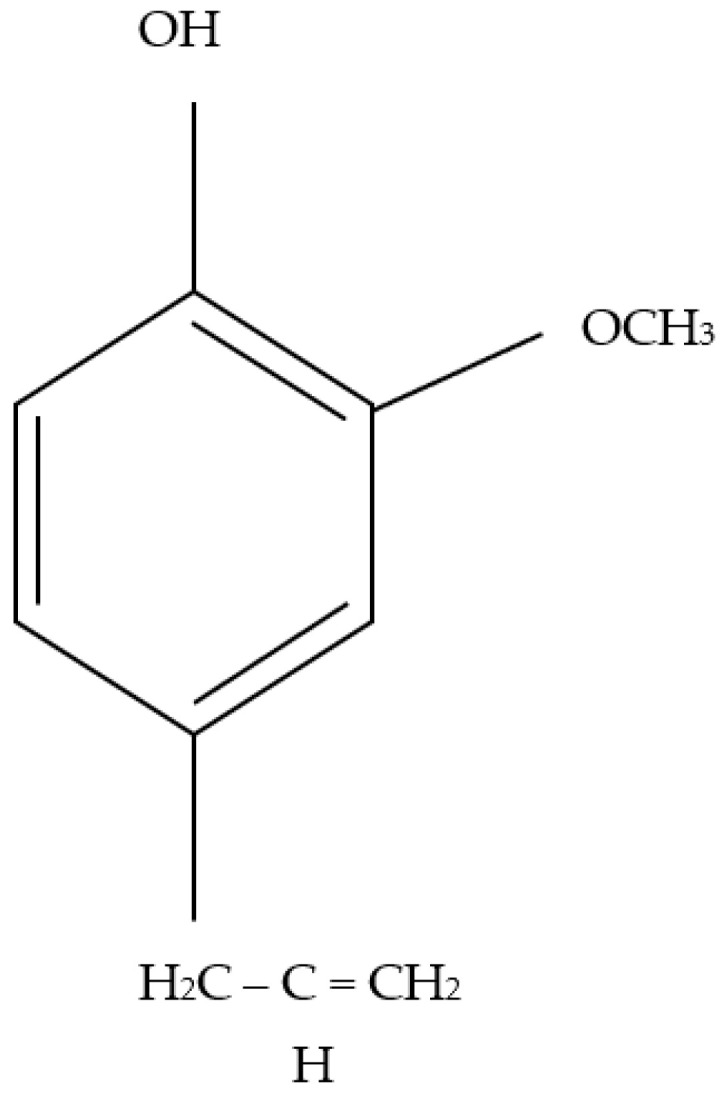
Structure of eugenol.

As eugenol (Figure 6) is the main component of clove bud oil [8], the clove bud oil (fibre) spectrum is characterised by eugenol peaks. These occur at 3520 cm^−1^ and corresponding to O-H bond stretching, 1606 and 1511 cm^−1^ are ascribable to C-C bond stretching vibrations in the phenyl ring, 1233 cm^−1^ corresponding to C-O bending, 1040 cm^−1^ is attributed to C-O bonds. The spectral bands and wavenumbers were comparable with results from similar work [47].

FTIR analysis thus confirmed the existence of clove bud oil in the fibres that were previously incorporated into the polymer solution and subsequently spun into fibres using pressurised gyration. This process was repeated for the other two oil containing fibres and their respective oils, alongside virgin PCL fibres, similar conclusions were drawn.

Clove bud oil containing fibres did prove to have antimicrobial activity, the FTIR interpretation presented here can confirm the inclusion of clove bud oil into the polymeric fibres. However, limitations of FTIR spectroscopy exist as it is neither able to objectively confirm the presence of oils nor provide information on the quantity of oil present in the fibres. It is therefore not possible to determine whether the used method of insertion of oils without extraction or additional/different solubility processes ensured the maximum quantity of oils available in the fibres. The FTIR spectrum can also not reveal whether the specific active components of the oil, that are assumed to be antimicrobial, are present in either the pure oil or the oil-containing fibres. However, after having obtained the final antimicrobial results where only a minimal difference was noted between the pure clove bud oil and the clove bud oil fibres, it can be deduced that the fibres indeed did possess the oil and its antimicrobial properties.

### 3.2. Antimicrobial Testing

**Figure 7:**
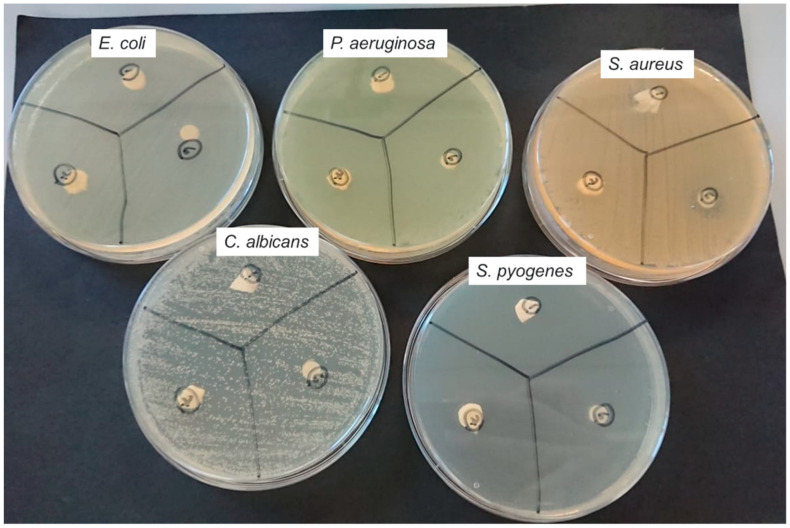
Petri dishes containing results of antimicrobial inhibition zone of clove bud oil fibres, pure clove bud oil, and virgin PCL fibres against the fungi and bacteria: *E. coli*; *P. aeruginosa*; *S. aureus*; *C. albicans*; *S. pyogenes*.

Clove bud oil fibres (C2) presented highly beneficial antimicrobial properties against four of the tested bacterial and fungal colonies. They showed a large zone of inhibition, especially when the original diameter of the sample is considered. The images in Figure 7 present the results of inhibition zone testing of clove bud oil fibres, pure clove bud oil, and pure PCL against: *E. coli*, *P. aeruginosa*, *S. aureus*, *C. albicans*, and *S. pyogenes*.

The disk diffusion test determines the antimicrobial effect of the samples. Whilst standardisation of the tests and direct comparison is not possible in this case due to differences in conditions and preparation, conclusions about the results can solely be interpreted. Interpretative standardisation also varies according to the antimicrobial sample used. Filter paper in standardised attempts of the test had a diameter of 6 mm, more than the diameter of the sample of this research (5 mm) [48]. Weight of the sample also differs but as the inhibition diameter highly exceeds the sample diameter, the results are concluded as particularly efficient. For *E. coli*, the diameter of inhibition lies at 18 mm. In comparison to the antimicrobial sample diameter, that represents 360%. For *P. aeruginosa*, the bacteria colony was completely resistant to clove bud oil showing no zone of inhibition. For *S. aureus*, the growth inhibiting effect extended to 220%, 11 mm, of the sample diameter. For *C. albicans*, the largest inhibition zone diameter measured was obtained against *C. albicans* of 30 mm, representing 600% of the sample diameter. For *S. pyogenes*, the inhibition zone diameter was measured at 20 mm, 400% in comparison to the sample diameter.

**Figure 8:**
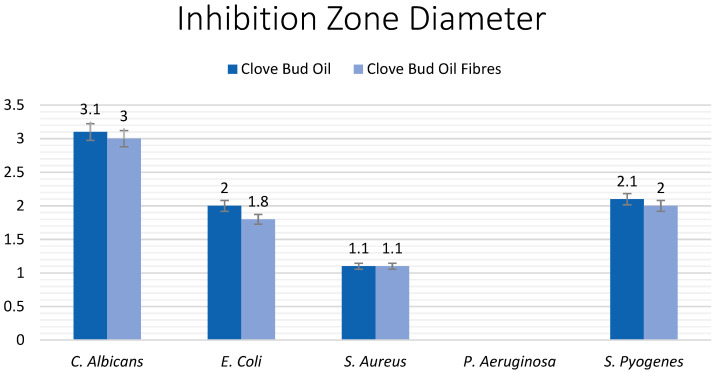
Inhibition zone diameters (cm) of clove bud oil and clove bud oil containing fibres against the fungus *C. albicans* and the bacteria *E. coli*, *S. aureus*, *P. aeruginosa*, and *S. pyogenes*.

Moreover, the comparison between the (filter paper) pure clove bud oil and the clove bud oil fibres (C2) provided a highly positive result. There existed only minor to no differences in inhibition zone diameter against all fungal and bacterial colonies. The differences are compared in Figure 8 and are given in percentages corresponding to 3.3%, 10.5%, 0%, and 4.9% against *C. albicans*, *E. coli*, *S. aureus*, and *S. pyogenes*, respectively. This further advocates for the very significant advantage of using antimicrobial dressings, as it negates the possibility of decreased effect in fibre form. It also validates that pressurised gyration is a facile and viable method of producing antimicrobial oil-containing fibres.

The antimicrobial effect of the clove bud oil is known to be due to the presence of its principle bactericidal component, which is known as eugenol [49]. Eugenol contains a phenolic chemical structure (Figure 6) and exhibits a high level of activity against a range of microorganisms. It does this because it has the ability to pass through the cell walls of Gram-positive bacteria, which causes cell wall degradation, damage to essential proteins and enzymes, leakage of cellular components, and death of the bacteria due to cell lysis [8]. Although other compounds do exist in addition to eugenol, the majority of the antimicrobial activity can be ascribed to eugenol; needless to say that the addition of other compounds would contribute to the antimicrobial abilities seen in this study [50]. As seen with other plant-based sources such as *Panax ginseng*, there exists a collection of bioactive compounds such as ginsenoside Rd and saponins [51,52]. The extract of just a single isolated compounds may not yield the complete set of benefits as seen here and in work by others; for this reason, the incorporation of plant-based extracts into reproducible bandage-like fibres is a procedure that could lead to many improvements in safe and sustainable approaches to wound healing. 

## 4. Conclusions

This work has shown the antimicrobial properties of oils derived from plants that are known to relieve various illnesses. It was shown that clove bud oil was successfully incorporated into bandage-like fibres that could be used as potential wound dressings.

The clove bud oil bandages presented in this work proved to have strong antimicrobial activity. The high percentage of eugenol in the oil (72–90%) is believed to be responsible for the large observed inhibition zones against the fungus *C. albicans* as well as the bacteria *E. coli*, *S. aureus*, and *S. pyogenes*.

When examining the antimicrobial properties, clove bud oil fibres exhibited excellent suitability, the inhibition zones from the antimicrobial testing of pure clove bud oil fibres compared to clove bud oil were nearly identical (difference of 0–10.5%). The results shown here indicate the suitability and relevance for further and improved production of clove bud oil and other natural oil-containing fibres using pressurised gyration. Overall, the work confirms that the antimicrobial activity of an oil is not affected by the fibre form.

It can therefore be concluded that clove bud oil fibres could provide an effective alternative to drug-based wound-bandages, without the toxicity of chemical medicines and be able to provide more sustainability into health and wound care. The combination of bioactive compounds within the clove bud oil contributes to the antimicrobial and other pro-wound healing effects. In this work, the fibres were able to be produced in less than 30 s, demonstrating that pressurised gyration is a suitable method for producing such materials for healthcare applications.

## Data Availability

Supporting data are available on request, please contact authors for specific data.

## References

[1] Petrovska B.B. (2012). Historical review of medicinal plants’ usage. Pharmacogn. Rev..

[2] Kelly A., Ahmed J., Edirisinghe M. (2022). Manufacturing Cyclodextrin Fibers Using Water. Macromol. Mater. Eng..

[3] Shedoeva A., Leavesley D., Upton Z., Fan C. (2019). Wound Healing and the Use of Medicinal Plants. Evid. Based Complement. Alternat. Med..

[4] Yousaf S., Hanif M.A., Rehman R., Azeem M.W., Racoti A., Hanif M.A., Nawaz H., Khan M.M., Byrne H.J. (2020). Chapter 32—Indian Pennywort. Medicinal Plants of South Asia.

[5] Balekar N., Katkam N.G., Nakpheng T., Jehtae K., Srichana T. (2012). Evaluation of the wound healing potential of *Wedelia trilobata* (L.) leaves. J. Ethnopharmacol..

[6] Conti P., Caraffa A., Gallenga C.E., Ross R., Kritas S.K., Frydas I., Younes A., Di Emidio P., Ronconi G., Pandolfi F. (2021). Powerful anti-inflammatory action of luteolin: Potential increase with IL-38. Biofactors.

[7] Sharma A., Khanna S., Kaur G., Singh I. (2021). Medicinal plants and their components for wound healing applications. Future J. Pharm. Sci..

[8] Chaieb K., Hajlaoui H., Zmantar T., Kahla-Nakbi A.B., Rouabhia M., Mahdouani K., Bakhrouf A. (2007). The chemical composition and biological activity of clove essential oil, *Eugenia caryophyllata* (*Syzigium aromaticum* L. Myrtaceae): A short review. Phytother. Res..

[9] Jitender S., Anupama B., Goel S.P. (2012). *Eugenia caryophyllata* Thunberg (family Myrtaceae): A review. Int. J. Res. Pharm. Biomed. Sci..

[10] Moon S.-E., Kim H.-Y., Cha J.-D. (2011). Synergistic effect between clove oil and its major compounds and antibiotics against oral bacteria. Arch. Oral Biol..

[11] Mahulette A.S., Hariyadi, Yahya S., Wachjar A. (2020). Physico-chemical properties of clove oil from three forest clove accession groups in Maluku. IOP Conf. Ser. Earth Environ. Sci..

[12] El Ghallab Y., Al Jahid A., Jamal Eddine J., Ait Haj Said A., Zarayby L., Derfoufi S. (2020). *Syzygium aromaticum* L.: Phytochemical investigation and comparison of the scavenging activity of essential oil, extracts and eugenol. Adv. Tradit. Med..

[13] Marchese A., Barbieri R., Coppo E., Orhan I.E., Daglia M., Nabavi S.F., Izadi M., Abdollahi M., Nabavi S.M., Ajami M. (2017). Antimicrobial activity of eugenol and essential oils containing eugenol: A mechanistic viewpoint. Crit. Rev. Microbiol..

[14] Campbell J.L., Coyer F.M., Mudge A.M., Robertson I.M., Osborne S.R. (2017). *Candida albicans* colonisation, continence status and incontinence-associated dermatitis in the acute care setting: A pilot study. Int. Wound J..

[15] Nobile C.J., Johnson A.D. (2015). *Candida albicans* Biofilms and Human Disease. Annu. Rev. Microbiol..

[16] Márquez-Rodríguez A.S., Nevárez-Baca S., Lerma-Hernández J.C., Hernández-Ochoa L.R., Nevárez-Moorillon G.V., Gutiérrez-Méndez N., Muñoz-Castellanos L.N., Salas E. (2020). In Vitro Antibacterial Activity of *Hibiscus sabdariffa* L. Phenolic Extract and Its In Situ Application on Shelf-Life of Beef Meat. Foods.

[17] Cheng S., Clancy C.J., Checkley M.A., Handfield M., Hillman J.D., Progulske-Fox A., Lewin A.S., Fidel P.L., Nguyen M.H. (2003). Identification of *Candida albicans* genes induced during thrush offers insight into pathogenesis. Mol. Microbiol..

[18] Rasko D.A., Rosovitz M.J., Myers G.S.A., Mongodin E.F., Fricke W.F., Gajer P., Crabtree J., Sebaihia M., Thomson N.R., Chaudhuri R. (2008). The Pangenome Structure of *Escherichia coli*: Comparative Genomic Analysis of *E. coli* Commensal and Pathogenic Isolates. J. Bacteriol..

[19] Qadri F., Svennerholm A.-M., Faruque A.S.G., Sack R.B. (2005). Enterotoxigenic *Escherichia coli* in Developing Countries: Epidemiology, Microbiology, Clinical Features, Treatment, and Prevention. Clin. Microbiol. Rev..

[20] Fernandez J., Hilliard J.J., Morrow B.J., Melton J.L., Flamm R.K., Barron A.M., Lynch A.S. (2011). Efficacy of a New Fluoroquinolone, JNJ-Q2, in Murine Models of *Staphylococcus aureus* and *Streptococcus pneumoniae* Skin, Respiratory, and Systemic Infections. Antimicrob. Agents Chemother..

[21] Sadikot R.T., Blackwell T.S., Christman J.W., Prince A.S. (2005). Pathogen-host interactions in *Pseudomonas aeruginosa* pneumonia. Am. J. Respir. Crit. Care Med..

[22] Johansson L., Norrby-Teglund A. (2013). Immunopathogenesis of streptococcal deep tissue infections. Curr. Top. Microbiol. Immunol..

[23] Capoor M.R., Nair D., Deb M., Batra K., Aggarwal P. (2006). Resistance to erythromycin and rising penicillin MIC in *Streptococcus pyogenes* in India. Jpn. J. Infect. Dis..

[24] Mosti G., Mattaliano V., Partsch H. (2008). Influence of different materials in multicomponent bandages on pressure and stiffness of the final bandage. Dermatol. Surg..

[25] Okur M.E., Karantas I.D., Şenyiğit Z., Üstündağ Okur N., Siafaka P.I. (2020). Recent trends on wound management: New therapeutic choices based on polymeric carriers. Asian J. Pharm. Sci..

[26] Tobudic S., Kratzer C., Lassnigg A., Presterl E. (2012). Antifungal susceptibility of *Candida albicans* in biofilms. Mycoses.

[27] Wenzel R.P., Gennings C. (2005). Bloodstream infections due to *Candida* species in the intensive care unit: Identifying especially high-risk patients to determine prevention strategies. Clin. Infect. Dis..

[28] Pal S., Sayana A., Joshi A., Juyal D. (2019). *Staphylococcus aureus*: A predominant cause of surgical site infections in a rural healthcare setup of Uttarakhand. J. Family Med. Prim. Care.

[29] Hong X., Edirisinghe M., Mahalingam S. (2016). Beads, beaded-fibres and fibres: Tailoring the morphology of poly(caprolactone) using pressurised gyration. Mater. Sci. Eng. C.

[30] Heseltine P.L., Hosken J., Agboh C., Farrar D., Homer-Vanniasinkam S., Edirisinghe M. (2019). Fiber Formation from Silk Fibroin Using Pressurized Gyration. Macromol. Mater. Eng..

[31] Ahmed J., Gultekinoglu M., Bayram C., Kart D., Ulubayram K., Edirisinghe M. (2021). Alleviating the toxicity concerns of antibacterial cinnamon-polycaprolactone biomaterials for healthcare-related biomedical applications. MedComm.

[32] Ahmed J., Altun E., Aydogdu M.O., Gunduz O., Kerai L., Ren G., Edirisinghe M. (2019). Anti-fungal bandages containing cinnamon extract. Int. Wound J..

[33] Aydogdu O.M., Altun E., Ahmed J., Gunduz O., Edirisinghe M. (2019). Fiber forming capability of binary and ternary compositions in the polymer system: Bacterial cellulose–polycaprolactone–polylactic Acid. Polymers.

[34] Altun E., Aydogdu M.O., Togay S.O., Sengil A.Z., Ekren N., Haskoylu M.E., Oner E.T., Altuncu N.A., Ozturk G., Crabbe-Mann M. (2019). Bioinspired Scaffold Induced Regeneration of Neural Tissue. Eur. Polym. J..

[35] Rong D., Chen P., Yang Y., Li Q., Wan W., Fang X., Zhang J., Han Z., Tian J., Ouyang J. (2016). Fabrication of Gelatin/PCL Electrospun Fiber Mat with Bone Powder and the Study of Its Biocompatibility. J. Funct. Biomater..

[36] Domalik-Pyzik P., Morawska-Chochół A. (2022). Preliminary Results on Heparin-Modified Double-Layered PCL and PLA-Based Scaffolds for Tissue Engineering of Small Blood Vessels. J. Funct. Biomater..

[37] Prakasam M., Locs J., Salma-Ancane K., Loca D., Largeteau A., Berzina-Cimdina L. (2017). Biodegradable Materials and Metallic Implants—A Review. J. Funct. Biomater..

[38] Abdul Khodir W.K., Abdul Razak A.H., Ng M.H., Guarino V., Susanti D. (2018). Encapsulation and Characterization of Gentamicin Sulfate in the Collagen Added Electrospun Nanofibers for Skin Regeneration. J. Funct. Biomater..

[39] Biggemann J., Müller P., Köllner D., Simon S., Hoffmann P., Heik P., Lee J.H., Fey T. (2020). Hierarchical Surface Texturing of Hydroxyapatite Ceramics: Influence on the Adhesive Bonding Strength of Polymeric Polycaprolactone. J. Funct. Biomater..

[40] Khatami M., Varma R.S., Zafarnia N., Yaghoobi H., Sarani M., Kumar V.G. (2018). Applications of green synthesized Ag, ZnO and Ag/ZnO nanoparticles for making clinical antimicrobial wound-healing bandages. Sustain. Chem. Pharm..

[41] Nešporová K., Pavlík V., Šafránková B., Vágnerová H., Odráška P., Žídek O., Císařová N., Skoroplyas S., Kubala L., Velebný V. (2020). Effects of wound dressings containing silver on skin and immune cells. Sci. Rep..

[42] Heseltine P.L., Ahmed J., Edirisinghe M. (2018). Developments in pressurized gyration for the mass production of polymeric fibers. Macromol. Mater. Eng..

[43] Ahmed J., Gultekinoglu M., Edirisinghe M. (2020). Bacterial cellulose micro-nano fibres for wound healing applications. Biotechnol. Adv..

[44] Illangakoon E.U., Mahalingam S., Matharu K.R., Edirisinghe M. (2017). Evolution of Surface Nanopores in Pressurised Gyrospun Polymeric Microfibers. Polymers.

[45] Kuppan P., Sethuraman S., Krishnan U.M. (2013). PCL and PCL-gelatin nanofibers as esophageal tissue scaffolds: Optimization, characterization and cell-matrix interactions. J. Biomed. Nanotechnol..

[46] Liverani L., Lacina J., Roether J.A., Boccardi E., Killian M.S., Schmuki P., Schubert D.W., Boccaccini A.R. (2018). Incorporation of bioactive glass nanoparticles in electrospun PCL/chitosan fibers by using benign solvents. Bioact. Mater..

[47] Mohammed M.J., Al-Bayati F.A. (2009). Isolation and identification of antibacterial compounds from *Thymus kotschyanus* aerial parts and *Dianthus caryophyllus* flower buds. Phytomedicine.

[48] Hudzicki J. (2009). Kirby-Bauer disk diffusion susceptibility test protocol. Am. Soc. Microbiol..

[49] Kim E.-H., Kim H.-K., Ahn Y.-J. (2003). Acaricidal Activity of Clove Bud Oil Compounds against *Dermatophagoides farinae* and *Dermatophagoides pteronyssinus* (Acari:  Pyroglyphidae). J. Agric. Food Chem..

[50] Devi K.P., Nisha S.A., Sakthivel R., Pandian S.K. (2010). Eugenol (an essential oil of clove) acts as an antibacterial agent against *Salmonella typhi* by disrupting the cellular membrane. J. Ethnopharmacol..

[51] Kim W.-K., Song S.-Y., Oh W.K., Kaewsuwan S., Tran T.L., Kim W.-S., Sung J.-H. (2013). Wound-healing effect of ginsenoside Rd from leaves of *Panax ginseng* via cyclic AMP-dependent protein kinase pathway. Eur. J. Pharmacol..

[52] Zhang Y., Wang Y., Zhu X., Cao P., Wei S., Lu Y. (2017). Antibacterial and antibiofilm activities of eugenol from essential oil of *Syzygium aromaticum* (L.) Merr. & L. M. Perry (clove) leaf against periodontal pathogen *Porphyromonas gingivalis*. Microb. Pathog..

